# Deeply Inverted and Biphasic T-Waves of Wellens’ Syndrome: A Characteristic Electrocardiographic Pattern Not To Forget

**DOI:** 10.7759/cureus.22130

**Published:** 2022-02-11

**Authors:** Ghulam Mujtaba Ghumman, Swathi Yarlagadda, Ratika Dogra, Fnu Salman

**Affiliations:** 1 Internal Medicine, St. Vincent Mercy Medical Center, Toledo, USA

**Keywords:** coronary angiography, myocardial infarction, stenosis, left anterior descending coronary artery, inverted t-waves, electrocardiogram (ecg), wellens’

## Abstract

Wellens’ syndrome refers to specific electrocardiographic (ECG) abnormalities of deeply inverted T-waves in the precordial leads, mainly V1-V3, associated with critical stenosis of the proximal left anterior descending (LAD) coronary artery. Identifying this specific pattern on the electrocardiogram is important as emergent treatment can prevent life-threatening myocardial infarction. We present a case of Wellens’ syndrome that had a combination of inverted and biphasic T-waves patterns and where timely identification of the abnormal ECG pattern by the emergency physician and prompt intervention by the cardiology team prevented the development of myocardial infarction and hence permanent damage to the heart.

## Introduction

The Wellens syndrome describes a typical electrocardiogram (ECG) pattern of deeply inverted or biphasic T-waves in the precordial leads that is highly specific for critical stenosis of the proximal left anterior descending coronary artery. This syndrome is also referred to as left anterior descending (LAD) coronary T-wave syndrome [1]. These patients usually present with chest pain and have normal to slightly elevated cardiac enzymes, with the ECG findings of typical inverted or biphasic T-waves in the precordial leads. Identifying this specific pattern on ECG is crucial as these patients can go on to develop myocardial infarction [2]. We present a case of Wellens’ syndrome with a combination of inverted and biphasic T-waves where timely identification of abnormal ECG patterns by the emergency physician and prompt intervention by the cardiology team prevented the development of myocardial infarction and hence permanent damage to the heart.

## Case presentation

**Figure 1:**
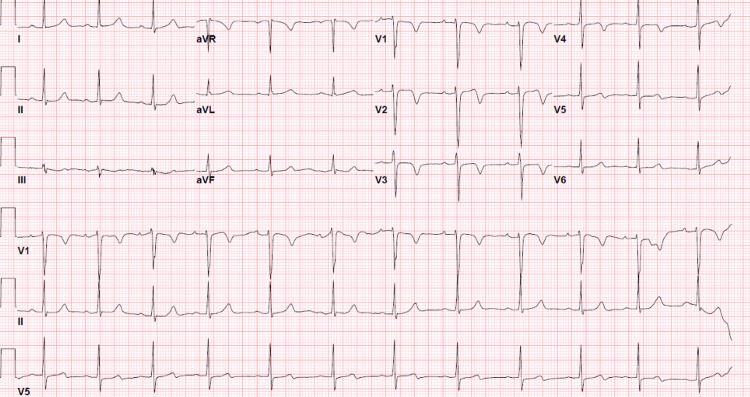
Deep T-wave inversions in leads V1-V3 and biphasic T-waves in leads V4-V5 without ST-segment abnormalities.

A 41-year-old African American female with a past medical history of medication-controlled hypertension presented with moderate to severe left chest pain radiating to the left arm associated with nausea and diaphoresis. Home medications included amlodipine and lisinopril. The patient reported having a few episodes of stress-related chest pain in the past, but she didn’t consider that significant hence didn’t seek medical attention. Her physical examination was unremarkable. ECG showed regular rate and rhythm with classic deep T-wave inversion in leads V1-V3 and biphasic T-wave in leads V4-V5 without ST-segment abnormalities (Figure 1). 

**Figure 2:**
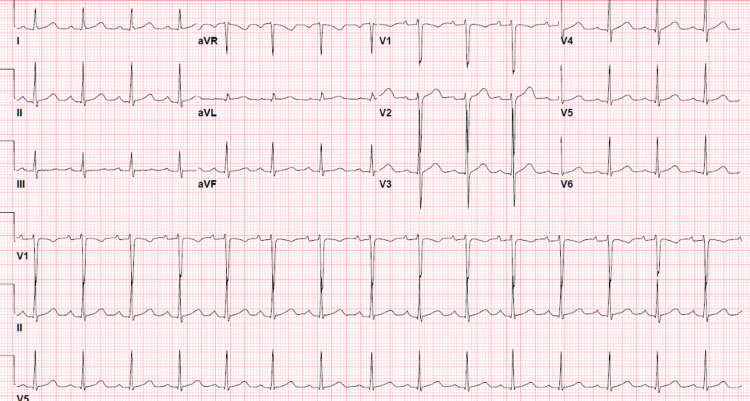
Normal EKG one month before presentation

EKG a month before the presentation was grossly normal (Figure 2).

**Figure 3:**
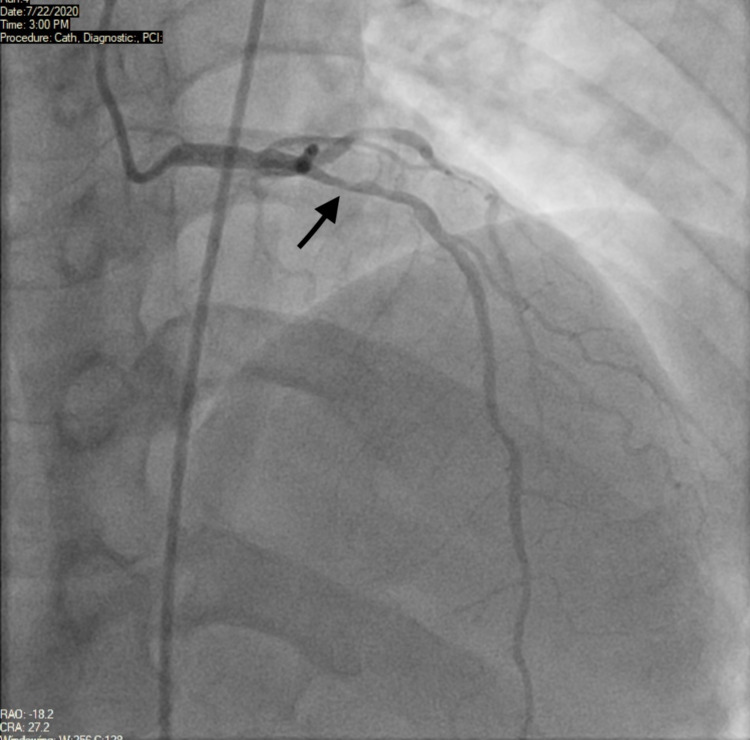
Left anterior oblique (LAO) view of angiography with cranial (CRA) angulation showing proximal stenosis of LAD (black arrow)

Initial high sensitivity troponins were 14 (reference range 0 to 14 ng/L) with a slight elevation to 18 and then 36, which trended down to normal. Complete blood count (CBC) and basic metabolic panel (BMP) were unremarkable. The patient received a loading dose of aspirin (325 mg) and was started on intravenous nitroglycerin and heparin. The cardiology team decided to proceed with coronary angiography (within one hour after arrival to the emergency department) that revealed 90% proximal and 70% mid LAD stenosis (Figure 3).

**Figure 4:**
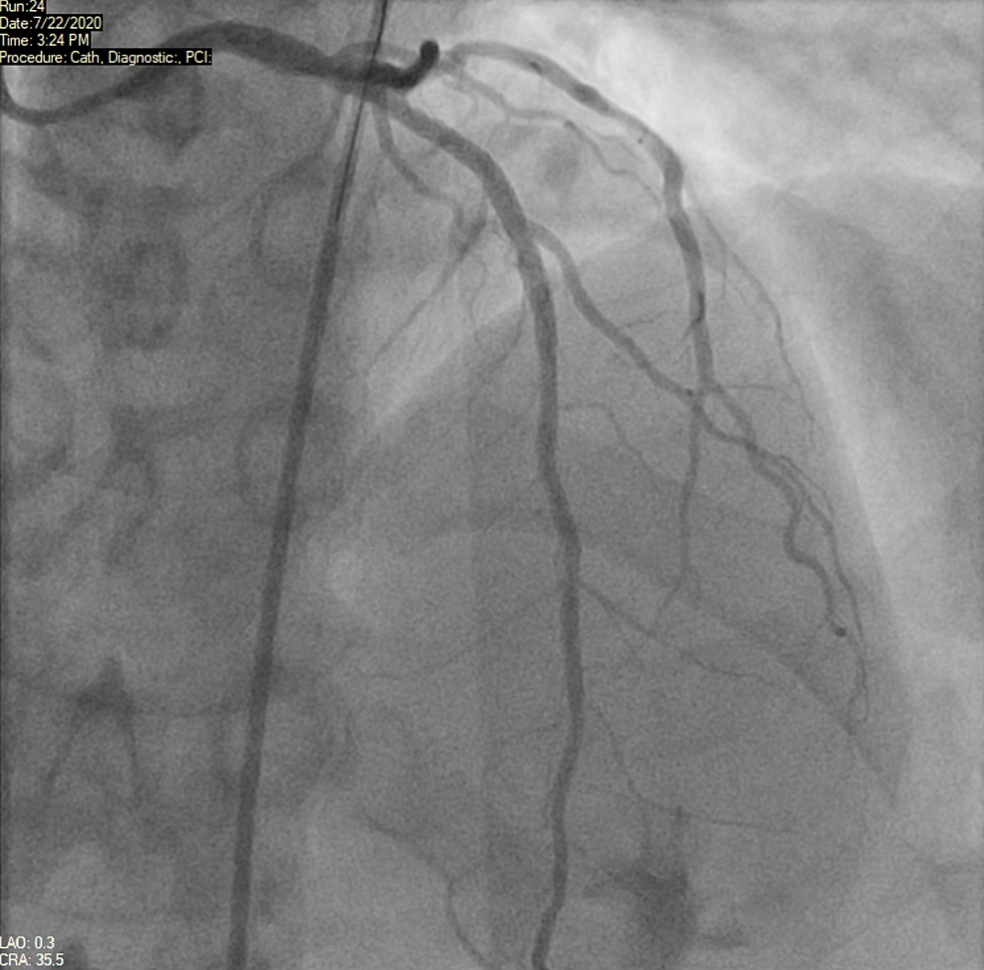
Left anterior oblique (LAO) view of angiography with cranial (CRA) angulation showing excellent grade 3 TIMI flow after stenting of LAD

Angioplasty was performed with two drug-eluting stents (DES) to LAD, reducing the stenosis to zero with good grade 3 TIMI (Thrombolysis in Myocardial Infarction) flow (Figure 4).

Echocardiography showed normal systolic and diastolic function with an ejection fraction of 55%. The patient was started on aspirin, ticagrelor, atorvastatin, and metoprolol tartrate and was discharged home in stable condition. 

## Discussion

The ECG pattern of Wellens’ syndrome is classified into type-A and type-B. Type A have deeply inverted T-waves (occurs in 75% of cases), and type B has biphasic T-waves (occurs in 25% of cases) in the precordial leads. However, some consider that one pattern can change to the other with ECG evolution in the same patient [3]. Wellens’ syndrome causes include an atherosclerotic plaque and increased oxygen demand with underlying risk factors of hypertension, hyperlipidemia, diabetes mellitus, smoking, metabolic syndrome, and increasing age [4]. It was first described by Wellens, De Zwaan, and colleagues in 1982 in patients consecutively admitted for unstable angina. Their study showed that a significant percent (26 out of 145 patients, i.e., 18%) of those patients have this specific ECG pattern in precordial leads, and 16 of those 18 patients (75%), despite being aggressively treated with nitroglycerin and beta-blocker went on to develop extensive anterior wall myocardial infarction within few weeks of admission [5]. Later, in 1989, they reported a prospective study, where they found that 180 out of 1260 (14%) admitted consecutively for unstable angina had this specific ECG pattern suggestive of LAD stenosis. One hundred eight patients had ECG abnormalities on admission, while the remaining 72 developed shortly thereafter. All those 180 patients had 50% or more narrowing (mean of 85%) of left anterior descending coronary artery on coronary angiography, with complete occlusion of LAD in 33 patients. Fifteen of those 180 patients had anterior wall myocardial infarction in the hospital (nine before and six during early revascularization). One hundred thirty-seven patients had abnormal systolic left ventricular wall motion, while the remaining 43 had normal systolic wall motion [2]. Gerson and colleagues had previously described a similar phenomenon in 1979 when they initially reported exercise-induced U-waves and later U-waves inversions at rest as a marker of left anterior descending coronary artery ischemia [6,7]. But the pattern is now more commonly known as Wellens’ syndrome, as also described by numerous authors in the subsequent years, likely because Wellens and the colleagues described it in detail and presented it correctly as T-wave abnormality instead of U-waves [8]. The diagnosis of Wellens syndrome is based mainly on ECG findings of deeply inverted or biphasic T-waves in Leads V2 and V3 (may also be seen in leads V1, V4, V5, and V6), without any significant ST segments elevation along with normal or slightly elevated cardiac enzymes. Wellens’ syndrome represents a pre-infarction state and is specific for critical stenosis of the left anterior descending coronary artery. There is a very high risk for the development of extensive myocardial infarction of the anterior cardiac wall and possibly death due to the unstable nature of the coronary perfusion [4]. A recent study showed that the T-wave abnormalities in the Wellens syndrome are associated with increased myocardial mechanical and electrical dispersion, likely contributing to infarction [9]. Therefore, early identification and prompt intervention are lifesaving in these cases. There is also a possibility of re-infarct even after the angioplasty due to stent re-stenosis of the coronary artery, so the patient must continue to take the antiplatelet medications with proper cardiology follow-up [10].

## Conclusions

The distinct ECG patterns of Wellens’ syndrome could be overlooked due to the absence of the typical ST-segment elevation as seen in ST-segment elevation myocardial infarction (STEMI). Therefore, the physicians need to be reminded of these typical ECG findings diagnostic for severe left anterior descending coronary artery stenosis with the risk of impending anterior wall myocardial infarction. Early recognition and prompt percutaneous coronary intervention prevent permanent heart damage.
